# MiR1885 Regulates Disease Tolerance Genes in *Brassica rapa* during Early Infection with *Plasmodiophora brassicae*

**DOI:** 10.3390/ijms22179433

**Published:** 2021-08-30

**Authors:** Parameswari Paul, Sushil Satish Chhapekar, Jana Jeevan Rameneni, Sang Heon Oh, Vignesh Dhandapani, Saminathan Subburaj, Sang-Yoon Shin, Nirala Ramchiary, Chanseok Shin, Su Ryun Choi, Yong Pyo Lim

**Affiliations:** 1 Department of Horticulture, College of Agriculture and Life Science, Chungnam National University, Daejeon 34134, Korea; parameswaripaul@gmail.com (P.P.); sushilchhapekar@gmail.com (S.S.C.); saijeevan7@gmail.com (J.J.R.); rederaser64@gmail.com (S.H.O.); vicky.bioinfo@gmail.com (V.D.); sami_plantbio86@yahoo.co.in (S.S.); 2 School of Biosciences, University of Birmingham, Birmingham B15 2TT, UK; 3 Department of Agricultural Biotechnology, Seoul National University, Seoul 08826, Korea; nogarded@snu.ac.kr (S.-Y.S.); cshin@snu.ac.kr (C.S.); 4 Interdisciplinary Program in Agricultural Genomics, Seoul National University, Seoul 08826, Korea; 5 School of Life Sciences, Jawaharlal Nehru University, New Delhi 110067, India; nrudsc@gmail.com; 6 Research Institute of Agriculture and Life Sciences, Seoul National University, Seoul 08826, Korea; 7 Plant Genomics and Breeding Institute, Seoul National University, Seoul 08826, Korea

**Keywords:** MicroRNA, TIR-NBS genes, QTL, R gene, *Brassica*, *Plasmodiophora brassicae*, disease resistance, clubroot, *B. rapa*

## Abstract

Clubroot caused by *Plasmodiophora brassicae* is a severe disease of cruciferous crops that decreases crop quality and productivity. Several clubroot resistance-related quantitative trait loci and candidate genes have been identified. However, the underlying regulatory mechanism, the interrelationships among genes, and how genes are regulated remain unexplored. MicroRNAs (miRNAs) are attracting attention as regulators of gene expression, including during biotic stress responses. The main objective of this study was to understand how miRNAs regulate clubroot resistance-related genes in *P. brassicae*-infected *Brassica rapa*. Two *Brassica* miRNAs, Bra-miR1885a and Bra-miR1885b, were revealed to target TIR-NBS genes. In non-infected plants, both miRNAs were expressed at low levels to maintain the balance between plant development and basal immunity. However, their expression levels increased in *P. brassicae*-infected plants. Both miRNAs down-regulated the expression of the TIR-NBS genes *Bra019412* and *Bra019410*, which are located at a clubroot resistance-related quantitative trait locus. The Bra-miR1885-mediated down-regulation of both genes was detected for up to 15 days post-inoculation in the clubroot-resistant line CR Shinki and in the clubroot-susceptible line 94SK. A qRT-PCR analysis revealed *Bra019412* expression was negatively regulated by miR1885. Both *Bra019412* and *Bra019410* were more highly expressed in CR Shinki than in 94SK; the same expression pattern was detected in multiple clubroot-resistant and clubroot-susceptible inbred lines. A 5′ rapid amplification of cDNA ends analysis confirmed the cleavage of *Bra019412* by Bra-miR1885b. Thus, miR1885s potentially regulate TIR-NBS gene expression during *P. brassicae* infections of *B. rapa*.

## 1. Introduction

Clubroot (CR), which is caused by the soil-borne pathogen *Plasmodiophora brassicae*, is a severe disease of crops in the family Brassicaceae. As a part of sustainable agriculture under deteriorating growth conditions due to long-term cultivation and climate change, the improvement of crops by introducing disease resistance traits is an important goal for plant breeders [1]. Traditionally, the introgression of new traits for crop improvement has been performed via interspecific and intraspecific hybridizations, and these methods have resulted in the successful generation of crop resources with resistance traits [2]. Because CR disease is a major source of economic losses [3], the CR resistance trait has been introduced into *Brassica* species using various resistance resources (primarily turnip, but other subspecies as well).

To fully utilize resistance traits, the mechanisms underlying their genetic regulation must be characterized. In the last two decades, genetic analyses of CR resistance have been conducted using a variety of resistant *Brassica rapa* germplasm. Additionally, the A genome of Brassicaceae species has been extensively investigated. Comparative analyses have revealed the high collinearity between the resistance loci in the genomes of other amphidiploids (with AB and AC genomes) and those in the A genome. Previous studies have identified the following 23 CR resistance loci distributed on seven chromosomes (A01, A02, A03, A05, A06, A07, and A08) in *B. rapa*: *CRa* [4,5], *CRaki* [6,7], *CRb* [8,9], *CRc, CRk* [10], *CRd* [11], *Crr1*, *Crr2* [12]), *Crr3* [13], *Crr4* [14], *CrrA05* [15], *CRs* [16], *PbBp3.1, PbBp3.3* [9], *qBrCR38-1, qBrCR38-2* [17], *Rcr1* [18], *Rcr2* [19], *Rcr4, Rcr8, Rcr9* [20], *Rcr3,* and *Rcr9^wa^* [21]. Chromosome A03 harbors at least 12 CR resistance loci (*CRa, CRaki, CRb, CRd, CRk, PbBp3.1, PbBp3.3, Crr3, Rcr1, Rcr2, Rcr4,* and *Rcr5*) effective against diverse pathotypes. After *CRa* was first identified at the *CR* locus in *B. rapa*, *CRb, CRk*, and *Crr3* were identified using diverse resistant materials and pathotypes. Recent advances in next-generation sequencing technology have enabled the easy, fast, and accurate identification of genomic regions related to qualitative and quantitative resistance traits [22].

Despite many genetic studies on resistance, relatively little is known about the candidate genes and the mechanisms controlling the CR resistance trait. Two loci related to CR resistance (*Crr1* and *CRa*) were discovered by map-based cloning, and TIR-NBS-LRR (TNL) genes were identified as candidate genes involved in resistance, based on gain-of-function analyses [5,23]. In the last decade, several loci (*CRd, Rcr1, Rcr2, Rcr4,* and *Rcr5*) were detected based on the identification of genome-wide variants through bulked segregant RNA sequencing and genotyping-by-sequencing [11,20,24,25]. These earlier studies identified candidate genes on the basis of abundant sequence variations between resistant and susceptible lines, as determined by high-throughput sequencing and analyses of functional similarity. Although different resistant materials and pathogens were used in these studies, the identified loci were localized on chromosome A03 and in genomic regions with a similar set of candidate genes.

Recent genomic, transcriptomic, and proteomic analyses have indicated that hormonal regulation, cell wall structure, secondary metabolites, and resistance genes (R genes) are involved in the CR resistance trait [26,27]. However, to characterize the mechanism regulating CR resistance, future studies should focus on the interrelationships among genes and how genes are regulated.

MicroRNAs (miRNAs) are small non-coding RNAs that are attracting attention as essential regulators of gene expression. They play a major role in the regulation of developmental and physiological processes [28,29] and in the expression of genes responsive to abiotic and biotic stresses, including R genes [30]. On the basis of the presence of specific domains, R genes are generally classified into five functionally distinct classes. The first class contains nucleotide-binding site–leucine-rich repeat (NBS-LRR) genes, which are further classified as Toll/interleukin 1 receptor (TIR)-NBS-LRR (TNL) and coiled-coil (CC)-NBS-LRR (CNL) genes. The second class comprises genes encoding transmembrane proteins, including receptor-like transmembrane proteins. The genes in the third class encode kinases, including serine–threonine kinases, whereas those in the fourth class encode kinases with receptor-like functions (e.g., receptor-like kinases). The fifth class contains atypical R genes [31]. At the molecular level, various R genes contribute to the direct or indirect recognition of pathogen-derived effectors that induce effector-triggered immunity, which frequently involves a series of responses, including the hypersensitive response (i.e., a type of programmed cell death) [32].

In recent decades, several studies have demonstrated the importance of miRNA-regulated RNA silencing for plant innate immunity. Initially, miR472 and miR482 were identified and experimentally confirmed to target various NLR genes [33,34]. In *Glycine max*, most R genes (178/290 CNLs and 171/235 TNLs) are directly regulated by miRNAs [35]. Additionally, miRNA target decoys (i.e., endogenous RNAs that can negatively regulate miRNA activity) have also been identified [36]. Subsequent studies on several plant species detected numerous miRNA families whose members target multiple R genes and are responsible for post-transcriptional gene silencing [18,37,38,39,40,41,42,43,44,45]. Such miRNAs are generally conserved in identical/similar species and their target sequences encode conserved R protein motifs [45]. For example, members of the miR482 superfamily specifically target a conserved P loop motif in NLR proteins that is crucial for function [46]. In tobacco, nta-miR6019 and nta-miR6020 are involved in the silencing of the N gene, encoding a TNL protein [42]. In *Medicago truncatula*, miR1510 is differentially expressed between the roots and nodules [47,48]. In a soybean, miR1510 cleaves TNL gene transcripts, thereby activating phased secondary small interfering RNA (phasiRNA) synthesis [37]. Similarly, miR482 and miR2118 initiate phasiRNA production from their NLR targets and function as the principal regulators of various genes encoding NLRs [42,43,45,49]. Interestingly, an invading pathogen can alter both the expression of R genes and miRNA-mediated R gene turnover [43,50]. In *B. rapa*, miR1885 regulates both an immune receptor gene (*BraTNL1*) and a growth-related gene (*BraCP24*) via the production of trans-acting small interfering RNAs [18].

The mechanisms underlying the interplay between a pathogen infection and the miRNA-mediated expression of R genes, especially those related to CR disease in *Brassica* species, are still poorly understood. Recent studies explored the role of long non-coding RNAs and mRNAs in *Brassica napus* [51,52] and *Brassica campestris* [17] responses to *P. brassicae*, but there are no reports describing the roles of miRNAs in the response of *B. rapa* to *P. brassicae*.

In this study, we investigated miRNAs that are structurally associated with a group of candidate genes related to CR resistance on chromosome A03. These genes have been consistently identified as being associated with stress resistance [19,20,25,53]. We predicted the targets of miR1885a and miR1885b in *B. rapa*. Further analyses revealed that both miR1885a and miR1885b are differentially expressed between resistant and susceptible genotypes, and these miRNAs negatively regulate R genes in response to pathogens. Under natural conditions, miR1885 expression is maintained at low levels to allow for normal plant development and basal immunity. Its expression levels peak after pathogen infections, indicative of the reallocation of energy between activities related to growth and immunity. Our findings provide insights into how gene expression is precisely controlled during the complex interaction between a pathogen and its host.

## 2. Results

### 2.1. Differences in the Responses of Resistant (CR Shinki) and Susceptible (94SK) B. rapa Inbred Lines to P. brassicae Pathotypes

**Figure 1:**
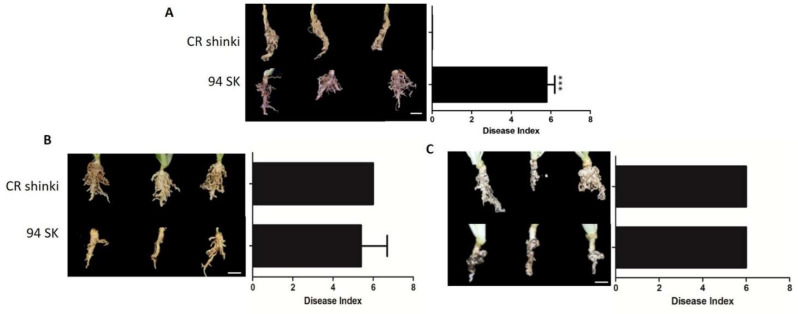
Differences in the responses of CR Shinki (resistant) and 94SK (susceptible) *Brassica rapa* inbred lines to three *Plasmodiophora brassicae* pathotypes. Disease symptoms on *B.* *rapa* roots infected with *P. brassicae* pathotypes (**A**) Race 4 [8], (**B**) Uiryeong, and (**C**) Banglim [54]. Disease severity was assessed according to the degree of root morphological modifications using the following rating scale: 0 = no symptoms; 1 = very slight swelling, usually confined to the lateral roots; 2 = a few small, separate globular clubs on the lateral roots; 3 = moderate clubbing on the lateral roots; 4 = large clubs on the lateral roots and slight swelling of the main root; 5 = larger clubs on the main root than on the lateral roots; 6 = severe clubbing on all roots. The mean disease index (*n* = 10) is presented to the right of the photos; the bar represents the standard error of three replicates. Asterisks indicate a significant difference at *p* < 0.001.

The responses of Chinese cabbage (*B. rapa*) lines to different *P. brassicae* pathotypes were compared on the basis of the disease index (DI). More specifically, the responses of CR Shinki and 94SK Chinese cabbage inbred lines to three well-known *P. brassicae* pathotypes (Race 4, Uiryeong, and Banglim) were analyzed [8,54] (Figure 1). In response to Race 4, the DI was significantly higher for the susceptible line (94SK) than for the resistant line (CR Shinki) (Figure 1A). In contrast to CR Shinki roots, the 94SK roots were severely damaged by CR. Both lines were similarly susceptible to pathotypes Uiryeong and Banglim (i.e., no significant difference in the DI) (Figure 1B,C). This suggests that CR Shinki exhibits pathotype-specific resistance, that is, it is resistant to only Race 4. Plants were photographed at 8 weeks post-inoculation. The DI values confirmed the resistance, and susceptibility, of the investigated Chinese cabbage lines to different *P. brassicae* pathotypes.

### 2.2. P. brassicae Infection Induces miR1885a and miR1885b Expression

**Figure 2:**
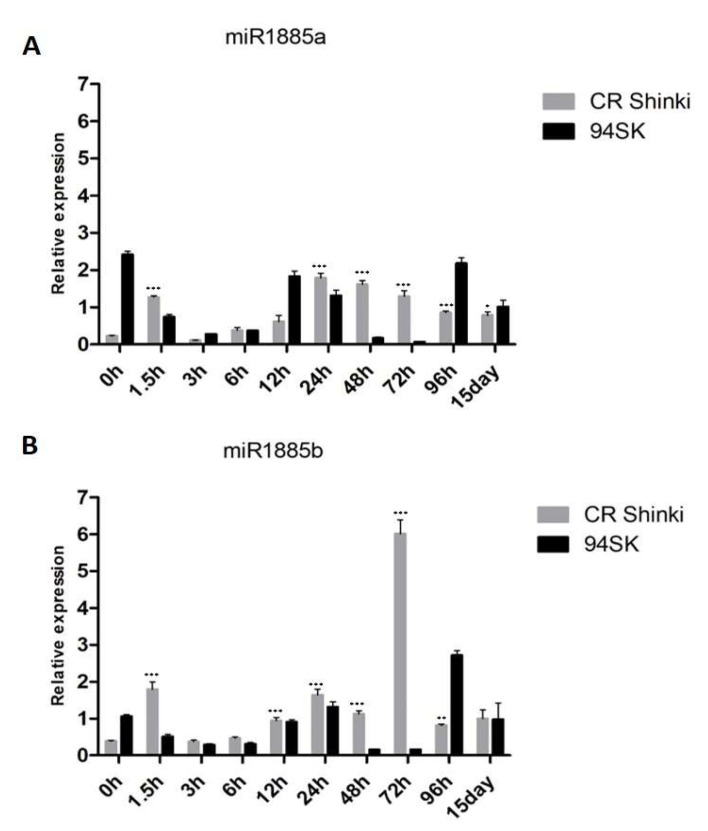
Expression of (**A**) miR1885a and (**B**) miR1885b in the roots of inbred lines CR Shinki (resistant) and 94SK (susceptible) inoculated with *P. brassicae*. The expression of both miRNAs increased significantly in CR Shinki post-inoculation (* *p* < 0.01, ** *p* < 0.05, and *** *p* < 0.001).

A 22-nucleotide miRNA, Bra-miR1885a, was initially identified as a *B. rapa* R gene-derived novel miRNA during a response to turnip mosaic virus (TuMV) [55]. It was subsequently revealed to be responsive to heat stress [56]. A recent study confirmed that Bra-miR1885 is induced in *B. rapa* and *B. napus* by TuMV, but not by any other pathogen [18]. These previous studies demonstrated that Bra-miR1885a precisely regulates plant growth and immunity in *Brassica* species, suggesting that this *Brassica*-specific miRNA may also play roles in responses to biotic stresses. In this study, miR1885 was induced in *B. rapa* infected with *P. brassicae* (Figure 2).

According to the miRBase database [57], the *Brassica* miR1885 family has the following two members: miR1885a (on chromosome 06: 25285096–25285118 [+]) and miR1885b (on chromosome 06: 25285163–25285185 [+]). The pre-miR1885 structure is presented in Supplementary Figure S1. The expression of both miR1885a and miR1885b in the root tissue of the resistant (CR Shinki) and susceptible (94SK) inbred lines before the inoculation with *P. brassicae* (0 h) as well as at 1.5 h, 3 h, 6 h, 12 h, 24 h, 48 h, 72 h, 96 h, and 15 days post-inoculation (Figure 2A,B) were investigated. At 1.5 h post-inoculation, the miR1885a and miR1885b expression levels increased significantly in CR Shinki, but decreased markedly in 94SK. In CR Shinki and 94SK, the expression levels of both miRNAs decreased at 3 h and 6 h post-inoculation but then increased at 12 h and 24 h. The miR1885a expression level remained steady in CR Shinki at 48 h and 72 h post-inoculation, but it decreased at these time-points in 94SK. At 96 h and 15 days post-inoculation, the miR1885a expression level remained steady in CR Shinki, whereas it increased in 94SK (Figure 2A). Regarding miR1885b, a slightly different expression pattern was observed from 48 h post-inoculation. Its expression level was higher in CR Shinki than in 94SK at 48 h, and it peaked in CR Shinki at 72 h at a level much higher than that in 94SK (Figure 2B). The miR1885b expression level was markedly lower in CR Shinki than in 94SK at 96 h, whereas there were no significant differences between the inbred lines at 15 days post-inoculation. Overall, the miR1885a and miR1885b expression levels were generally higher in CR Shinki than in 94SK following the inoculation with *P. brassicae*. Considered together, these findings suggest that Bra-miR1885 might contribute to the *B. rapa* response to *P. brassicae*.

### 2.3. Both miR1885a and miR1885b Target TIR-NBS Genes

The miRNA targets were predicted on the basis of several parameters, including complementarities with miR1885a and miR1885b sequences and the unpaired energy (UPE) required to unfold the target site. Using stringent criteria for target prediction (expectation value of up to 2 and UPE value of up to 25), 11 targets were predicted for miR1885b and five targets were predicted for miR1885a (Supplementary Table S1). Interestingly, gene ontology analyses indicated that most of the target genes were on chromosome A03 and encoded disease resistance proteins with TNL and TX structures. Twelve loci (*CRa*, *CRb*, *CRaki*, *PbBp3.1*, *PbBp3.2*, *CRk*, *CRd*, *Crr3*, *Rcr1*, *Rcr2*, *Rcr4*, and *Rcr5*) related to disease resistance are located on chromosome A03 [4,6,8,9,10,11,20,58,59]. The *CRb* resistance-related quantitative trait locus (QTL) region in CR Shinki is located at the basal end of chromosome A03, and several gene loci are located nearby. Even though these earlier studies were performed using different resistant resources/germplasm (Chinese cabbage, pakchoi, and turnip) for introgressing resistance loci as well as different pathotypes, they all consistently detected certain genes (*Bra019409*, *Bra019410*, *Bra019412*, and *Bra019413*) in this QTL region as candidates for the CR resistance trait [19,20,24,25,53,59]. Among the candidate genes within the QTL, *Bra019410*, *Bra019412*, and *Bra020936* had an expectation value of 2, which was calculated on the basis of the affinity of miRNAs for their targets. Interestingly, *Bra020936*, which is located in a different QTL region on chromosome A08 [20], was also predicted as a miR1885b target, but it was excluded from further analyses because CR Shinki lacks the corresponding resistance locus.

Our results indicated that miR1885b targets both *Bra019412* and *Bra019410* with strict complementarity (expectation value approximately 2) and high free energy values, whereas miR1885a only targets *Bra019412* with relatively low complementarity (expectation value approximately 5) (Supplementary Table S1). The prediction analysis indicated that miR1885a inhibits target genes via the ‘translation’ process, whereas miR1885b inhibits target genes via the ‘cleavage’ process. These findings suggest that miR1885b is mainly involved in the inhibition of target genes.

### 2.4. Structure of Proteins Encoded by Bra019412 and Bra019410

**Figure 3:**
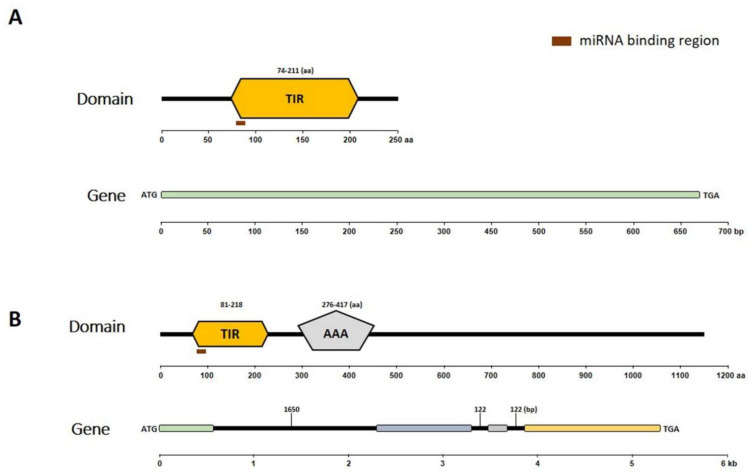
Analyses of conserved domains in putative proteins as well as gene structures. (**A**) *Bra019412* encodes only the TIR domain, whereas (**B**) *Bra019410* encodes the TIR and AAA domains.

Next, the conserved domains of the putative encoded proteins and gene structures of *Bra019412* and *Bra019410* (Figure 3) were identified. A Simple Modular Architecture Research Tool (SMART) analysis of the Bra019410 protein revealed the following two major domains: the TIR domain (amino acids 81 to 218) and the AAA domain (amino acids 276 to 417) (Figure 3B). A SMART analysis of the Bra019412 protein revealed only the TIR domain (amino acids 74 to 211) (Figure 3A). The exon–intron distribution of both genes was examined by comparing the coding and genomic sequences using the GSDS online software (Figure 3). This analysis detected three introns in *Bra019410*, but no introns in *Bra019412*. The characteristics of the putative proteins were determined using the ExPASy ProtParam online tool. The predicted molecular weights of Bra019410 and Bra019412 were 130.82 kDa and 247.88 kDa, respectively. The aliphatic index of Bra019410 was 93.35 and the grand average of hydropathicity (GRAVY) value was −0.22, which confirmed its hydrophilic nature. Similarly, the aliphatic index of Bra019412 was 81.67 and its GRAVY value was −0.228, indicating that it is also hydrophilic in nature. The isoelectric point of Bra019410 was 6.33, indicating it is an acidic protein, whereas that of Bra019412 was 8.74, indicating it is an alkaline protein.

### 2.5. Both miR1885a and miR1885b Negatively Regulate TIR-NBS Genes

**Figure 4:**
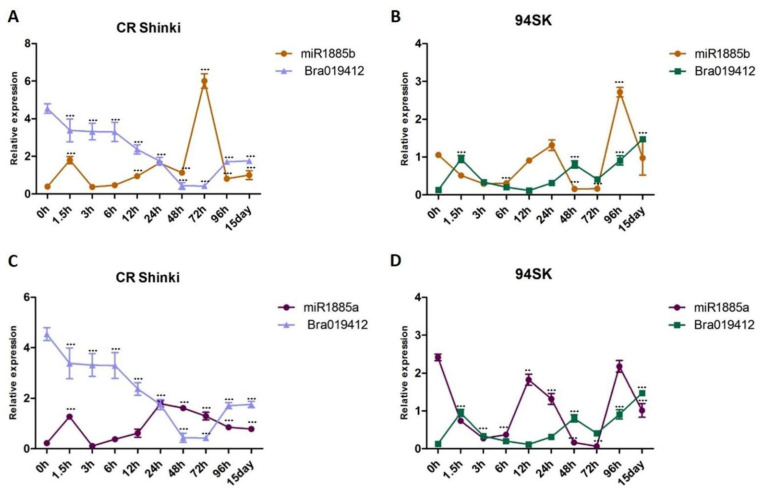
Analysis of the expression of *Bra019412* with miR1885b or miR1885a in the root tissues of *B. rapa* infected with *P. brassicae*. Correlation of the expression between (**A**) miR1885b and *Bra019412* in CR Shinki, (**B**) miR1885b and *Bra019412* in 94SK, (**C**) miR1885a and *Bra019412* in CR Shinki, and (**D**) miR1885a and *Bra019412* in 94SK as determined by qRT-PCR. The transcript level of the *B. rapa* actin gene served as the internal control for target gene expression, whereas the U6 snRNA level served as the internal control for mature miRNA expression. The miRNA and gene expression levels over time in inoculated plant samples were normalized against the corresponding expression levels in untreated plants. For each time-point, more than six individual plants were pooled (*n* > 6) and independent experiments were replicated three times. Standard error bars are presented for each time-point. Asterisks indicate a significant difference at *** *p* < 0.001.

The correlation between the expression levels of miRNAs and the transcript levels of both target genes (*Bra019412* and *Bra019410*) was investigated. As the miR1885b expression level increased from 1.5 h onwards, it down-regulated *Bra019412* expression until 15 days post-inoculation, indicating that miR1885b acts as a negative regulator of *Bra019412* in the resistant CR Shinki line (Figure 4A). In the susceptible 94SK line, miR1885b also appears to function as a negative regulator, although the *Bra019412* transcript level was lower in 94SK than in CR Shinki (Figure 4B). The miR1885a expression level in CR Shinki also increased at 1.5 h post-inoculation, but it was not as high as the miR1885b expression level and it was not correlated with the down-regulation of *Bra019412*, implying that miR1885a might be a partial negative regulator (Figure 4C). Additionally, miR1885a down-regulated the expression of *Bra019412* in 94SK, but only at the early post-inoculation time-points (up to 12 h). The expression pattern of miR1885a was dissimilar to that of miR1885b (Figure 4D).

**Figure 5:**
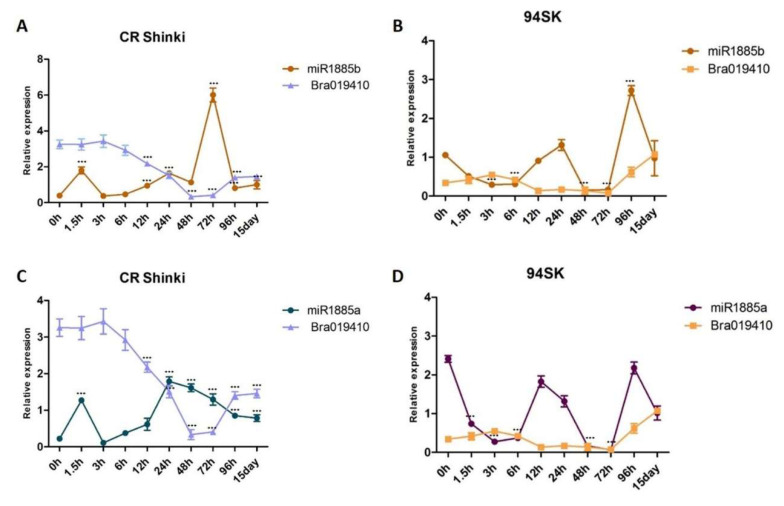
Analysis of the expression of *Bra019410* and miR1885b or miR1885a in the root tissues of *B. rapa* infected with *P. brassicae*. Correlation of the expression between (**A**) miR1885b and *Bra019410* in CR Shinki, (**B**) miR1885b and *Bra019410* in 94SK, (**C**) miR1885a and *Bra019410* in CR Shinki, and (**D**) miR1885a and *Bra019410* in 94SK, as determined by qRT-PCR. The transcript level of the *B. rapa* actin gene served as the internal control for target gene expression, whereas the U6 snRNA level served as the internal control for mature miRNA expression. The miRNA and gene expression levels, over time, in inoculated plant samples were normalized against the corresponding expression levels in untreated plants. For each time-point, more than six individual plants were pooled (*n* > 6) and independent experiments were replicated three times. Standard error bars are presented for each time-point. Asterisks indicate a significant difference at *** *p* < 0.001.

The target analysis with stringent settings predicted that miR1885b targets *Bra019410*. However, the expression analysis demonstrated that miR1885b affects *Bra019410* expression only at post-inoculation time-points after 3 h (Figure 5). In CR Shinki, the *Bra019410* transcript level was stable until 3 h post-inoculation, even though miR1885b expression was up-regulated (Figure 5A,C). However, at the later time-points, there was a negative correlation between miR1885b levels and the *Bra019410* transcript levels. This result suggests that miR1885b only partially controls *Bra019410* expression (Figure 5A,C). In 94SK, there were no correlations between miR1885b or miR1885a levels and *Bra019410* transcript levels (Figure 5B,D), suggesting that both miRNAs may only partially control *Bra019410* expression.

### 2.6. Expression Patterns of miR1885b, Bra019412, and Bra019410 in Multiple Resistant and Susceptible Lines

**Figure 6:**
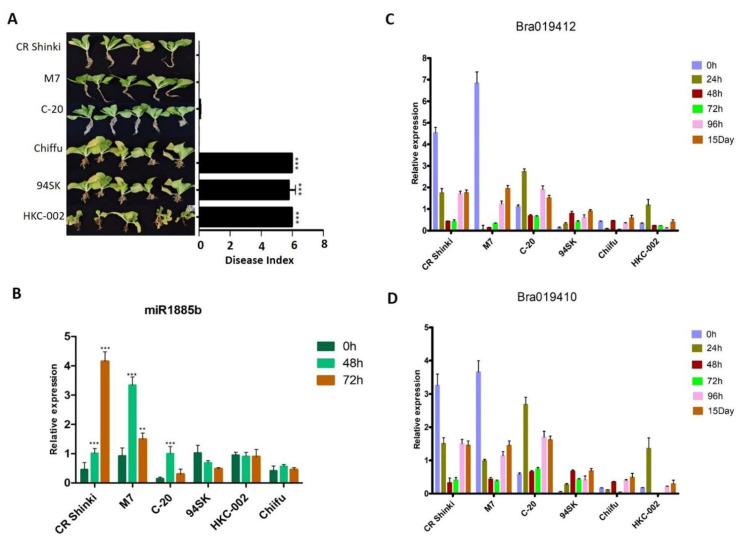
Expression patterns of miR1885b, *Bra019412*, and *Bra019410* in multiple resistant and susceptible lines. (**A**) Effects of the *P. brassicae* Race 4 infection of resistant (CR Shinki, M7, and C-20) and susceptible (94SK, HKC-002, and Chiifu) genotypes with the same alleles. Disease symptoms were examined at 8 weeks post-inoculation. For each line, the disease index, calculated according to the disease severity, is provided to the right of the photos. Bars represent the standard error. Significant differences are indicated with asterisks (** *p* < 0.05, and *** *p* < 0.001). Expression of (**B**) miR1885b, (**C**) *Bra019412*, and (**D**) *Bra019410* in resistant (CR Shinki, M7, and C-20) and susceptible (94SK, HKC-002, and Chiifu) genotypes. For each time-point, the expression levels were calculated on the basis of three independent replicates.

To confirm the miR1885b, *Bra019412*, and *Bra019410* expression patterns in CR-resistant and CR-susceptible genotypes, their transcript levels in multiple genotypes with contrasting responses to CR were investigated. On the basis of a DI analysis, three resistant genotypes (CR Shinki, M7, and C-20) and three susceptible genotypes (94SK, Chiifu, and HKC-002) (Figure 6A) were selected. After the inoculation with *P. brassicae*, miR1885b expression increased significantly in all resistant lines (particularly at 48 h) (Figure 6B). However, there were no significant changes in expression in the susceptible lines, suggesting miR1885b may be involved in the regulation of disease development in the resistant lines (Figure 6B). Overall, the root *Bra019412* and *Bra019410* transcript levels were significantly higher in the three resistant lines than in the susceptible lines (Figure 6C,D). The *Bra019412* transcriptional pattern was similar in CR Shinki and M7; the transcript levels gradually decreased from 0 h to 48 h post-inoculation (except at 12 h in M7), but then increased at 96 h and 15 days post-inoculation (Figure 6C). The *Bra019412* transcript levels in C-20 also generally decreased over the first 48 h post-inoculation (except at 24 h), but then increased at 96 h and 15 days post-inoculation, which was consistent with the expression pattern in the other two resistant lines (CR Shinki and M7). The expression profiles indicate that 24–96 h post-inoculation is a critical period for the regulation of resistance. The *Bra019412* transcript levels were lower in the susceptible lines than in the resistant lines at all time-points, with the exception of 24 h in HKC-002 (Figure 6C).

The *Bra019410* transcriptional patterns in the resistant and susceptible lines were similar to those of *Bra019412* (Figure 6D). In general, the *Bra019410* transcript levels post-inoculation were higher in the resistant lines than in the susceptible lines. The *Bra019410* transcript level gradually decreased from 0 h to 48 h post-inoculation in CR Shinki (except at 3 h) and M7 (Figure 6D, Figure 4A), but then increased at 96 h and 15 days post-inoculation. The *Bra019410* and *Bra019412* transcriptional patterns were identical in C-20 (Figure 6C). The *Bra019410* transcript levels were lower in the susceptible lines than in the resistant lines, except at 24 h (i.e., HKC-002).

### 2.7. Confirmation of miR1885b-Mediated Cleavage of Bra019412 by 5′ Rapid Amplification of cDNA Ends (RACE)

**Figure 7:**
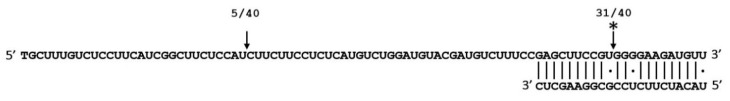
5′ RACE analysis revealing the cleavage site in regions targeted by miR1885b in the roots of infected CR Shinki plants. The miR1885b and *Bra019412* sequences are aligned and the predicted cleavage site is indicated with an arrow and an asterisk. The numbers above the sequence indicate the identified cleavage site in independent clones.

To confirm whether the decrease in the target gene transcript level was due to miR1885b/miR1885a-guided cleavage, the 5′ end of the cleaved *Bra019412* sequences was analyzed by 5′ RACE (Figure 7). The RNA extracted from the roots and leaves was subjected to a 5′ RACE analysis to identify the cleavage products of the target gene. In the root samples, a cleavage site (12th nucleotide) was detected in the miRNA-binding region of the target gene, confirming that *Bra019412* is a target of miR1885b (Figure 7).

A second cleavage site 47 bases upstream of the predicted cleavage site was also detected. Regarding the RNA extracted from the leaves, no cleavage was detected at the predicted cleavage site of the target gene. The 5′ RACE analysis confirmed that cleavage products were derived from the interaction between the target gene and miR1885b, but not miR1885a. Thus, only miR1885b contributes to mRNA degradation and CR resistance.

## 3. Discussion

Clubroot is a devastating disease of *Brassica* crops worldwide. The CR resistance trait is reportedly controlled by various genes at both dominant (mostly in *B. rapa*) and recessive loci. Although numerous CR resistance-related loci/genes have been identified over the last few decades, the associated gene expression and regulation remain relatively uncharacterized. MicroRNAs are vital post-transcriptional regulators of R genes, including those related to disease resistance. Therefore, in the current study, the regulation of R gene expression by miRNAs, in Chinese cabbage lines infected with *P. brassicae*, was investigated. In the absence of pathogen infections, the detection of self-antigens by R proteins typically leads to autoimmunity and adversely affects plant survival. Accordingly, maintaining a balance between defense responses and plant development is critical. In plants, to minimize fitness costs, it is essential that R protein activities are stringently regulated [38,60,61]. The miRNA-mediated regulation (or turnover) of R genes allows plants to control the potential fitness consequences in the absence of pathogen infections. Thus, coordinating disease resistance and yield has become a key objective in plant breeding. Several miRNAs targeting R genes have been identified in various plant species in the last decade [35,41,42,43]. However, the roles of these miRNAs in plant defense responses and how they are regulated during pathogen infections are still unexplored in plants, in general, but especially in *Brassica* crops. Previously, we conducted a genome-wide investigation of miRNAs from *B. rapa* [62]. Additionally, Kim et al. [56] investigated heat stress-responsive miRNAs in *B. rapa*. To the best of our knowledge, the present study is the first to analyze the miRNA roles related to CR resistance in *B. rapa*.

Clubroot decreases the productivity of *Brassica* crops worldwide. A previous study on *B. napus* revealed the differential expression of miRNAs in plants infected with *P. brassicae* as well as several miRNAs targeting genes related to auxin signaling and transcription factors for hormone homeostasis during disease development [51]. Recently, Zhu et al. [17] analyzed long non-coding RNA and mRNA profiles after a *P. brassicae* infection of pakchoi (susceptible line) to elucidate the molecular basis of pathogenesis. The long non-coding RNAs responsive to *P. brassicae* in both resistant and susceptible genotypes were reported by Summanwar et al. [52], and several *P. brassicae*-responsive long non-coding RNAs that mainly target genes on chromosome A08 were identified.

In this study, miR1885, which is associated with CR resistance, was characterized. Initially, miR1885 was identified as an R gene-derived novel miRNA in *B. rapa* by He et al. [55] and Kim et al. [56]. It has since been confirmed to contribute to the regulation of the resistance to TuMV [18]. On the basis of sequence complementarity, miRNAs interact with their target genes to initiate their regulatory functions. The target genes of miR1885 were predicted using a plant-specific psRNA target prediction program [63]. Further analyses confirmed that miR1885 binds to the predicted targets in the region encoding the TIR domain. The TIR domain plays a vital role in the recognition of pathogen effectors [32,64]. It is a signaling domain involved in inducing cell death and it plays a major role in basal defense mechanisms [65]. Nandety et al. [66] proved that the TIR domain alone is sufficient for pathogen recognition.

Genes at the *CRb* locus were predicted to be specific targets of miR1885b. Various studies conducted worldwide using different genetic materials and without shared resources identified several gene loci at *CRb* and adjacent regions [19,20,25,53,58,59]. These studies revealed the importance of this locus for CR resistance and consistently identified a common set of genes, including *Bra019409*, *Bra019410*, *Bra019412*, and *Bra019413*.

Although these earlier studies identified various candidate R genes, the underlying regulatory mechanisms were not elucidated. Shivaprasad et al. [43] demonstrated that the miRNA-mediated silencing of disease resistance-related mRNAs in plants infected with viruses and bacteria ultimately leads to the pathogen-induced expression of genes encoding NBS-LRR defense-related proteins. They also identified a member of the miR483/2118 superfamily as the master regulator of disease resistance in tomato. Similarly, in this study, the role of miR1885, which targets *Bra019412*, in disease resistance was investigated. A structural analysis indicated *Bra019412* consists of a single exon and encodes a protein with only a TIR domain. MiR1885 also targets *Bra019410*, but its expression was not perfectly negatively correlated with *Bra019410* transcript levels. Consequently, further analyses were focused on the control of *Bra019412* expression. To understand the differences in defense mechanisms between inbred lines resistant and susceptible to CR, *B. rapa* plants were infected with *P. brassicae* and then gene expression levels at different time-points were analyzed. Consistent with the findings of an earlier transcriptome analysis of a segregating F_1_ population containing the *Rcr1* locus [25], *Bra019412* was differentially expressed between the CR-resistant (overall up-regulation) and CR-susceptible (down-regulation) lines. Our results indicate that the observed diversity in the expression patterns was at least partly due to miRNAs.

**Figure 8:**
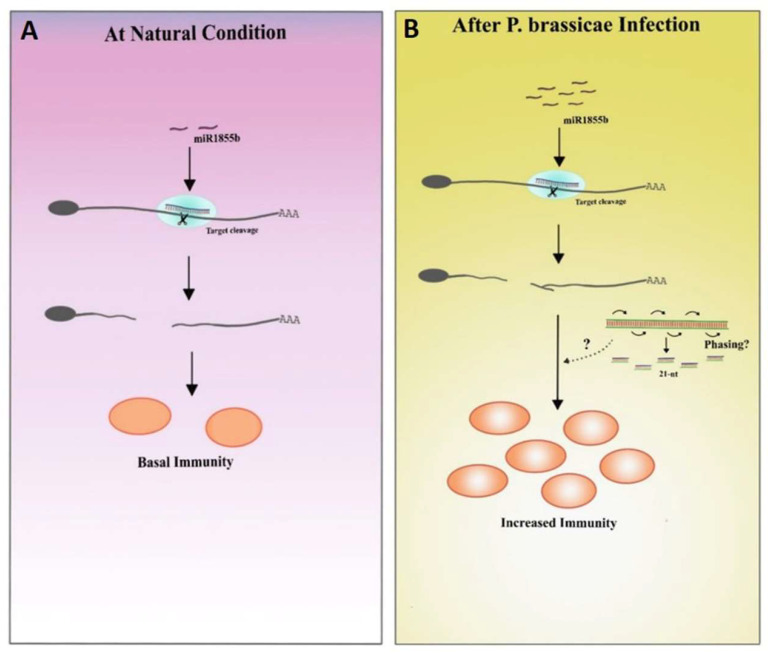
Proposed hypothesis based on the results of this study. (**A**) Under natural conditions, miR1885b is expressed at low levels, which is conducive to basal immunity. (**B**) During pathogen infections, miR1885b levels increase, which may enhance immunity through phasiRNAs.

In the current study, miR1885 was expressed at low levels under control conditions to maintain basal active immunity and *Bra019412* was stably transcribed to facilitate normal plant development (Figure 8A). The miR1885 levels increased only during the pathogen infection (Figure 8B). These results imply that a low miR1885 level is required for basal defense. Similar to our findings, Zou et al. [5] reported that Arabidopsis miR172b, which increases in abundance during seedling development, indirectly promotes the transcription of the gene encoding the immune receptor FLS2 through the post-transcriptional silencing of *TOE1* and *TOE2*, which encode suppressors of *FLS2* transcription. In another study on *B. rapa* infected by TuMV, an increase in miR1885 levels promoted precursor processing, suggesting that a TuMV infection increases the accumulation of miRNA-processing proteins [18].

In the present study, target gene expression increased substantially only in response to the Race 4 pathogen, consistent with the idea that each resistance locus has a different effect on CR development [9,20]. Shivaprasad et al. [43] noted that many genes encoding NBS-LRR proteins are associated with race-specific effector-triggered immunity. Additionally, *Bra019412* may interact with these genes and activate the associated disease resistance genes/clusters during pathogen infections, ultimately leading to quantitative resistance [67,68,69,70]. The TIR domain of Bra019412 likely acts as a link between the pathogen and the signaling function of the R protein [65,66,71]. Our results demonstrate that *Bra019412* plays a major role only in *CRb*-mediated CR resistance, which is consistent with the findings of previous studies, in which *Bra019412* was identified as a defense response-related candidate gene at a CR resistance locus in *B. rapa* [20,25,59]. Our observations suggest that the miRNA-mediated active regulation of immune receptors plays dynamic roles in the modulation of plant immunity.

MicroRNAs targeting NBS-LRR genes have distinct characteristics enabling them to target highly conserved motifs. Thus, they may regulate the expression of multiple members of gene families. This involves evolutionarily conserved interactions between small RNAs and their targets [55,72]. In the present study, miR1885b specifically targeted a gene encoding a protein with a TIR domain (Figure 3). The expression of TIR/TIR-NBS genes may be regulated by miRNAs in various ways. For example, the target sites may be expanded or lost and/or there may be feedback regulation between miRNAs and their target genes (Park and Shin, 2018). Recently, Cui et al. (2020) demonstrated that miR1885 targets NBS-LRR genes to activate phasiRNA generation when there is an excess of NBS-LRR proteins, indicative of self-regulation. This type of gene regulation prevents the undesirable production of TIR-NBS-LRR proteins. Similarly, we revealed that miR1885 regulates R gene turnover, thereby affecting disease resistance possibly through trans-acting RNA silencing [18]. Other studies confirmed the involvement of miR1885a in heat stress responses [56] and TuMV resistance [18]. This is the first report describing the role of miR1885b in a biotic stress response (i.e., CR disease).

## 4. Materials and Methods

### 4.1. Plant Materials and Pathogen Inoculation

*Brassica rapa* CR Shinki (CR resistant) and 94SK (CR susceptible) plants were grown in a growth chamber for 4 weeks and then inoculated with three *P. brassicae* pathotypes (Race 4, Uiryeong, and Banglim). Inbred line CR Shinki is resistant to the Race 4 pathotype [8], but susceptible to the Uiryeong (unpublished data) and Banglim [54] pathotypes. Plant samples were collected at two different infection stages. Samples for the initial infection stage were collected at 1.5, 3, 6, 12, 24, 48, 72, and 96 h post-inoculation, whereas samples for the late infection stage were collected at 15 days post-inoculation. The collected samples were immediately frozen in liquid nitrogen and then stored frozen until used for the gene expression analysis.

A *P. brassicae* suspension (2 × 10^6^ CFU mL^−1^) was used to infect plants via irrigation (i.e., injected into the soil). Briefly, a 5 mL aliquot of the spore suspension was added to the soil around 4-week-old plants. The differences in gene expression were verified in additional CR-resistant inbred lines (M7 and C-20) and CR-susceptible inbred lines (Chiifu and HKC002). The CR symptoms on plants were evaluated at 5 weeks post-inoculation as described by Choi et al. [54]. The DI was calculated according to a 0–6 scale, with 0 indicating the absence of disease symptoms and 6 indicating severe gall formation all over the roots. Ten plants per line were inoculated, and the experiments were performed in triplicate.

### 4.2. Prediction of the Target Genes of miR1885a and miR1885b

The target genes of miR1885a and miR1885b were predicted using psRNA Target [63]. This software predicts targets based on the reverse complementarity between miRNAs and target transcripts using the proven scoring scheme. It also evaluates the target site accessibility by calculating the UPE required for unwinding the secondary structure around the miRNA target site on the mRNA. The data were collected and then manually checked to remove repeated or irrelevant information. Finally, targets with an E-value < 4, a UPE < 25, and with significant matches in the seed region (7/8 for the second to eighth bases and 3/5 for the 12th to 16th bases from the 5′ end of the miRNA) were selected.

### 4.3. Validation of miRNA and Target Gene Expression by a qRT-PCR Analysis

Total RNA was isolated from the leaves and roots of plants at different growth stages using the Plant RNeasy kit (Qiagen, Hilden, Germany). The RNA quality and quantity were estimated by gel electrophoresis and by using a NanoDrop spectrophotometer (Agilent, Santa Clara, CA, USA). The miRNA expression levels were verified by stem-loop qRT-PCR. The forward and reverse stem-loop primers for each miRNA were designed and synthesized (Bioneer, Daejeon, Korea) (Table S1). First-strand cDNA was synthesized from 1 µg RNA using the SuperScript III First-Strand Synthesis System (Invitrogen, Carlsbad, CA, USA); the manufacturer-recommended procedure was slightly modified. To increase the efficiency of the reverse transcription, a pulsed reverse transcription reaction was completed using the following PCR program: 16 °C for 30 min, 60 cycles of 30 °C for 30 s, 42 °C for 30 s, and 50 °C for 1 s (Varkonyi-Gasic et al. 2007). The reverse transcriptase was inactivated by incubating the mixture at 85 °C for 5 min. The qRT-PCR analysis was performed using the QuantiSpeed SYBR Kit (PhileKorea, Seoul, Korea) and the CFX96 Touch Real-Time PCR Detection System (Bio-Rad, Berkeley, CA, USA). The PCR program was as follows: 95 °C for 3 min, 40 cycles of 95 °C for 15 s, 58 °C for 20 s, and 72 °C for 15 s. Immediately after the final PCR cycle, a melting curve analysis was conducted (increase from 65 °C to 95 °C in increments of 0.2 °C) to check the PCR product specificity. The reactions were completed in triplicate, and the experiment was repeated at least twice. A control reaction without the template and reverse transcriptase was included for each miRNA. The *B. rapa* U6 snRNA gene served as the internal reference control.

To analyze target gene expression, cDNA was synthesized from 1 µg RNA using the Topscript RT DryMIX kit (Enzynomics, Daejeon, Korea). The qRT-PCR analysis was performed in triplicate using the QuantiSpeed SYBR Kit (PhileKorea, Seoul, Korea) and the CFX96 Touch Real-Time PCR Detection System (Bio-Rad). The PCR program was as follows: 95 °C for 10 min, 40 cycles of 95 °C for 15 s, and 60 °C for 1 min. Immediately after the final PCR cycle, a melting curve analysis was conducted (increase from 65 °C to 95 °C in increments of 0.2 °C). Data were acquired during the annealing/extension step and were analyzed using the CFX manager software (version 2.1) (Bio-Rad, CA, USA). The *B. rapa* actin gene served as the internal reference control. The primers used to amplify the miRNA and gene sequences are listed in Supplementary Table S2. The comparative Ct method (2^−ΔΔCt^) was used to quantify the relative miRNA and target gene expression levels (Livak and Schmittgen 2001). Significant differences were detected using Student’s *t*-test. The miRNA and target gene expression levels were quantified using three replicates. Mean miRNA and target gene expression levels were normalized against the U6 snRNA and actin gene expression levels, respectively.

### 4.4. Target Gene Validation by 5′ RACE

The predicted targets were validated by 5′ RACE using a SMARTer RACE Kit (Clontech Laboratories, Takara Korea Biomedical Inc., Seoul, Korea). Briefly, 1 µg total RNA samples extracted from the roots and leaves of CR Shinki and 94SK plants infected with *P. brassicae* as well as non-infected (control) plants were used for the 5′ RACE assay. For the infected samples, the RNA samples extracted from plants at different infection stages were pooled. These total RNA samples were ligated with SMARTer II A oligonucleotides from the SMARTer RACE Kit and then the RNA was reverse transcribed using the supplied CDS Primer. Next, a PCR amplification was performed twice, using the long universal primer mix (UPM) and gene-specific primers in the first reaction and the short UPM and nested gene-specific primers in the second reaction. The PCR amplification was performed using 2× Advantage Taq Pre-mix. Amplified PCR products were purified and ligated into pRACE vectors using an In-Fusion HD Cloning Kit (Clontech, Laboratories, Takara Korea Biomedical Inc., Seoul, Korea). An AccuPrep^®^ Gel Purification Kit (Bioneer, Daejeon, Korea) was used to purify the target PCR products. The ligated products were inserted into competent cells. Plasmids were isolated from individual transformed clones using an AccuPrep^®^ Plasmid Mini Extraction Kit and then sequenced (Bioneer, Daejeon, Korea). The sequences were analyzed using BioEdit software version 7.2.

## 5. Conclusions

The current study demonstrated that miR1885a and miR1885b are differentially expressed between CR-resistant and CR-susceptible *B. rapa* genotypes. These miRNAs target TIR-NBS genes, specifically *Bra019412* and *Bra019410*. Overall, the target genes were expressed at higher levels in the resistant plants than in the susceptible plants. A negative correlation between miR1885b and *Bra019412* expression was detected, and a 5′ RACE analysis confirmed the cleavage of *Bra019412* by miR1885b. These findings revealed that miR1885b is critical for CR tolerance/resistance because it regulates *Bra019412* expression, especially in response to a *P. brassicae* infection. Future research should clarify how miR1885b regulates gene expression in plants infected with *P. brassicae*. The findings of this study are relevant for future investigations of miRNA-based regulation of CR development in *B. rapa* and related species. Additionally, the data presented herein will form the basis of the future functional characterization of the miRNAs, controlling the expression of disease resistance genes, in important vegetable and oilseed crops.

## Data Availability

All the necessary data generated are provided in the form of figures, tables and supplementary information.

## Supplementary Materials

The following are available online at www.mdpi.com/article/10.3390/ijms22179433/s1: Figure S1: Bra-miR1885 pri-miRNA structure, Table S1: Predicted targets of miR1885a and miR1885b, and Table S2: List of the primers used in this study.
