# Supplementary figures and images for: MiR1885 Regulates Disease Tolerance Genes in Brassica rapa during Early Infection with Plasmodiophora brassicae
Source: Int J Mol Sci. 2021 Aug 30;22(17):9433. doi: 10.3390/ijms22179433 (PMC8430504; doi:10.3390/ijms22179433)

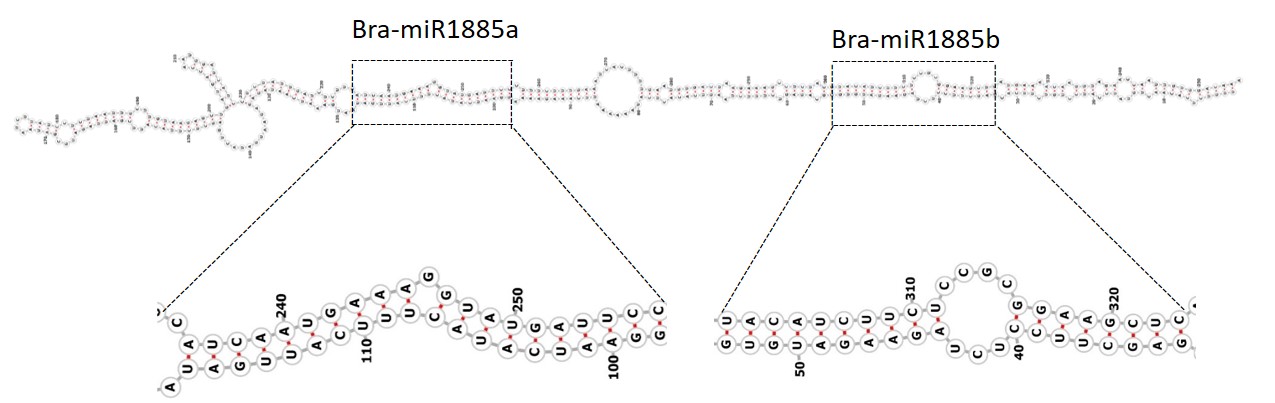

Supplement: Supplementary file 1 [file ijms-22-09433-s001.zip › suppl data/Suple Fig1.jpg]
